# Predictors of language proficiency and cultural identification in heritage bilinguals

**DOI:** 10.3389/fcomm.2022.994709

**Published:** 2022-08-31

**Authors:** Sayuri Hayakawa, Ashley Chung-Fat-Yim, Viorica Marian

**Affiliations:** 1 Department of Communication Sciences and Disorders, Northwestern University, Evanston, IL, United States; 2 Department of Psychology, Oklahoma State University, Stillwater, OK, United States

**Keywords:** vocabulary knowledge, cultural identification, proficiency, native language, heritage speakers, bilingualism

## Abstract

According to the 2020 U.S. Census Bureau, more than 66 million residents over the age of 5 in the United States speak a language other than English at home. Some bilinguals become dominant in the majority language that is spoken in the community as opposed to their native “heritage” language acquired at home. The objective of the current study was to uncover the predictors of language proficiency and cultural identification in different groups of heritage speakers. In our sample, heritage speakers acquired their heritage language first and English second and rated their proficiency in their heritage language lower than in English. We found that English proficiency was most reliably predicted by the duration of heritage language immersion, while heritage language proficiency was most reliably predicted by contexts of acquisition and exposure to both languages. Higher heritage language proficiency was associated with greater heritage language experience through friends and reading, less English experience through family, and later age of English acquisition. The trade-off between heritage language and English language experience was more pronounced for non-Spanish than Spanish heritage speakers. Finally, despite higher proficiency in English, cultural identification was higher with the heritage language, and was predicted by heritage language receptive proficiency and heritage language experience through family and reading. We conclude that self-reported proficiency and cultural identification differ depending on heritage speakers’ native languages, as well as how the heritage language and majority language are acquired and used. Our findings highlight the importance of taking individual language history into consideration when combining different groups of heritage speakers.

## Introduction

A growing percentage of the U.S. population speaks a language other than English at home. From 23.06 million in 1980 (Zeigler and Camarota, 2019) to 66.09 million in 2020 (U.S. Census Bureau, 2020), the number of people over the age of 5 who speak a non-English language at home has nearly tripled. These non-English home languages are often referred to as heritage languages and carry familial, cultural, and historical significance. Heritage bilinguals tend to feel strong personal connections to their heritage culture. However, as a result of acquiring the majority language at an early age and being formally educated in the majority language, heritage bilinguals generally prefer using the language of the community as opposed to their home language(s) (Valdés, 2000; Scontras et al., 2015).

Heritage bilinguals vary greatly in the age of second language acquisition and heritage language proficiency. While some heritage bilinguals immigrate to the host country with their parents and acquire the majority language in early childhood at school, others are born in the host country to foreign-born parents and acquire both languages simultaneously. Furthermore, while some heritage bilinguals have native-like proficiency in both languages, others show better linguistic command in the majority language than home language. Some can communicate fluently in both languages but are unable to read and write in the heritage language, and others have some understanding of the heritage language but have limited expressive skills (Montrul, 2005). Thus, heritage bilinguals are qualitatively distinct from second-language learners and native monolingual speakers (see Montrul, 2011 for review). Given that heritage speakers exist along a continuum of linguistic abilities and experiences, the present study aims to capture the linguistic predictors associated with self-reported measures of proficiency and cultural identification in different groups of heritage bilinguals.

### Language proficiency

There are several factors impacting heritage language proficiency, including language exposure (Gathercole and Thomas, 2009; Hoff et al., 2012; Thomas et al., 2014; Gollan et al., 2015; Jia and Paradis, 2015; Unsworth, 2016; Hovsepian, 2018; Makarova et al., 2019; Giguere and Hoff, 2020; Tao et al., 2021; Vorobyeva and Bel, 2021) and frequency of use (Hakuta and D’Andrea, 1992; Bedore et al., 2012; Albirini, 2014; Chen et al., 2018; Schmid and Yilmaz, 2018; Daskalaki et al., 2019; Otwinowska et al., 2021) in and outside of the home. Access to a heritage language community that extends beyond the home context positively predicts heritage language vocabulary and lexical retrieval (Albirini, 2014; Gollan et al., 2015; Schmid and Yilmaz, 2018; Tao et al., 2021), morphosyntax (Kupisch and Rothman, 2018; Rodina et al., 2020; Torregrossa et al., 2022), and pronunciation (Au and Romo, 1997; de Leeuw et al., 2010; Stoehr et al., 2017; Karayayla and Schmid, 2019; McCarthy and de Leeuw, 2022). Being surrounded by native speakers of the heritage language affords opportunities to listen and practice the language in various settings and discuss a wide variety of topics. Furthermore, heritage bilinguals exist along a continuum of linguistic abilities. In terms of reading and writing, heritage bilinguals are more likely to be literate in the majority language by virtue of being educated in that language. If heritage bilinguals do become literate in the heritage language, their reading skills tend to be better than their writing skills (Polinsky, 2015). Therefore, home and socio-linguistic contexts play important roles in the development of heritage language proficiency, and heritage bilinguals often exhibit variable degrees of fluency depending on the type of linguistic ability under examination (e.g., listening, speaking, reading, and writing).

In addition to the frequency of heritage language use, the age of second language acquisition and duration of immersion have been found to predict heritage language proficiency. The later a child becomes exposed to the majority language, the more likely they are to attain and retain competency in their heritage language (Polinsky and Kagan, 2007; Albirini, 2014; Jia and Paradis, 2015; Montrul, 2016; Gharibi and Boers, 2017; Meir et al., 2017; Armon-Lotem et al., 2021; Meir and Janssen, 2021). Studies have shown that sequential bilinguals often have greater proficiency in their heritage language than simultaneous bilinguals (e.g., Jia and Aaronson, 2003; Carreira and Kagan, 2011). For instance, children who acquire the majority language simultaneously or soon after the heritage language (e.g., before the age of 3) often score lower on tests of HL vocabulary (Gharibi and Boers, 2017; Armon-Lotem et al., 2021) and morphosyntax (Albirini, 2014; Jia and Paradis, 2015; Meir et al., 2017; Meir and Janssen, 2021) relative to children who spent more time learning the heritage language before acquiring the majority language. Age of acquisition predicts language aptitude and preference even among bilinguals who acquire the majority language later in adolescence. In a longitudinal study, Jia and Aaronson (2003) evaluated the changes in language preferences and Chinese proficiency among native Chinese-speaking children and adolescents who immigrated to the United States. Participants who immigrated to the United States at an early age (before the age of 9) switched their language preference from Chinese to English and became more proficient in English than Chinese within the first year. Those who immigrated to the United States at a later age (between 10 and 16 years of age) maintained their preference for Chinese across all 3 years and continued to use Chinese with their parents and siblings. Altogether, such findings demonstrate that both age of second language acquisition and duration of immersion influence heritage language proficiency.

To determine which factors promote heritage language proficiency, Gollan et al. (2015) tested Chinese-English and Spanish-English heritage bilingual adults on the Multilingual Naming Test (MINT; Gollan et al., 2012), which is an *objective* measure of language proficiency. For Chinese-English bilinguals, higher heritage language proficiency was associated with exposure to a greater number of heritage speakers during childhood. For Spanish-English bilinguals, higher heritage language proficiency was instead associated with less English use. The authors proposed that the differences between groups may stem from cross-cultural variations in the interpretation of the questionnaire items. Across all participants, proficiency in the heritage language was uniquely predicted by the number of heritage language speakers encountered during childhood, the primary caregiver’s level of English proficiency, and the participants’ age of English acquisition. For Persian-English bilingual children, parents’ attitude toward the heritage language was the strongest predictor of heritage language proficiency (as measured by a verbal fluency task and auditory picture-word matching test) in simultaneous bilinguals, whereas the age at emigration was the strongest predictor of heritage language proficiency in sequential heritage bilinguals (Gharibi and Boers, 2017). These findings demonstrate that individual variation within and across different groups of heritage bilinguals influences heritage language proficiency. The present study thus compares Spanish-English heritage speakers to other groups of heritage speakers (i.e., non-Spanish) living in the United States on heritage language and English proficiency ratings.

### Cultural identification

Language serves as a bridge for creating a sense of belonging to an ethnic group in children (Yu, 2015; Arredondo et al., 2016), adolescents (Phinney et al., 2001; Oh and Fuligni, 2010), and adults (Noels et al., 1996; Cho, 2000; Chen et al., 2008; Gatbonton and Trofimovich, 2008; Yu, 2015). Across all ages, greater proficiency in a heritage language is associated with stronger ethnic identity and affiliation with the ethnic group. However, heritage speakers vary in their cultural affiliation toward their heritage language and majority language. For example, individuals from minority groups sometimes report conflicting identities, in which they want to preserve the cultural values associated with their heritage language, but also want to fit in with the culture associated with the majority language (Phinney, 1990). On open-ended questions from the Multigroup Ethic Identity Measure and Ethnic Identity Scale, Arredondo et al. (2016) found that Spanish-English heritage bilingual children reported feeling a sense of pride for being able to speak Spanish, showed an appreciation for cultural diversity, enjoyed communicating exclusively with friends and family in a “secret” language, and expressed positivity toward helping their parents learn English and in turn, learning Spanish from their parents. In the same study, some of the children described Spanish as confusing or too difficult at times. Furthermore, heritage bilinguals are more likely to assimilate to the customs and practices of the host culture with each successive generation compared to the last (Felix-Ortiz et al., 1994). Hence, among heritage bilinguals, factors related to heritage language proficiency and migration, such as age of second language acquisition and duration of immersion, may predict cultural identification.

In a large heterogeneous sample of adult bilinguals varying in language and cultural backgrounds, Schroeder et al. (2017) identified the linguistic factors that predict cultural identification. Increased first language (L1) exposure through media, higher L1 proficiency, fewer years immersed in a second language (L2) family context, but more years immersed in an L2 school/work context led to increased first-language cultural affiliation. In contrast, increased immersion in an L2 school/work context, lower L2 perceived accent, and earlier L2 age of acquisition was associated with increased cultural identification with the second-language culture. These findings demonstrate that factors related to the second language influence both first-language and second-language cultural affiliation, whereas factors associated with the first language only influence first-language cultural identification. These effects also differed by age of L2 acquisition and whether the language was learned in a formal or informal context. Schroeder et al. argued that through language, bilinguals can access their culture by interacting with members of the same cultural group, actively participating in various cultural activities, and engaging in media from that culture (through TV, radio, and books). To our knowledge, no study to date has taken a similar approach in identifying linguistic predictors of cultural identification among different groups of heritage bilinguals.

With over 40.5 million people over the age of 5 speaking Spanish at home, Spanish is by far the most spoken non-English language in the United States (U.S. Census Bureau, 2020). Therefore, Spanish heritage bilinguals may have more opportunities to use and practice with native speakers and engage in cultural activities with members of the same cultural group than other non-English heritage bilinguals. For example, in the city of Chicago, Latinos are the second largest ethnic group at 29.7%, whereas Asians and other cultural groups make up around 12.7% of the city’s population (The Economist, 2017). Second, the one-to-one mapping between the Spanish language and Latino culture is less clear, as multiple cultural groups from various countries speak Spanish. In contrast, the mapping between language and culture for other languages is more consistent (e.g., Korean with Korea). Hence, there is the possibility that the linguistic and cultural experiences of Spanish heritage bilinguals are more diverse and less homogeneous compared to non-Spanish heritage bilinguals. Third, studies have shown that the motivation for maintaining the heritage language differs between Spanish-English heritage learners and non-Spanish heritage learners. Hur et al. (2021) examined the expectations and attitudes toward heritage language courses. While Spanish-English heritage learners perceived their classes as a necessary tool for professional success, Korean-English heritage learners used their classes as a way to reconnect with their Korean culture and other members of their heritage language community. For these reasons, we distinguish between Spanish and non-Spanish heritage bilinguals to examine how predictors of proficiency and cultural identification are moderated by native language background.

### The present study

The goal of the present study was to identify the predictors of self-reported language proficiency and cultural identification in different groups of heritage speakers. Specifically, we examined how age of acquisition, duration of immersion, and contexts of acquisition and exposure (i.e., through friends, family, media, reading, and language tapes and self-instruction) influenced self-reported measures of proficiency and cultural identification in the heritage language and in English among Spanish heritage bilinguals and non-Spanish heritage bilinguals. Considering heritage bilinguals typically have better comprehension than oral skills in their heritage language (Polinsky, 2015), we separated expressive (speaking) from receptive (understanding and reading) proficiency in our analyses. Based on past research, we hypothesized that heritage language proficiency and cultural identification will be predicted by heritage language usage in informal contexts, such as in the home through family and in the community through friends (Gollan et al., 2015; Jia and Paradis, 2015; Montrul, 2016), as well as the age of English acquisition and length of immersion in an English-speaking country (Montrul, 2008; Gathercole and Thomas, 2009; Vorobyeva and Bel, 2021). In addition, we hypothesized that English proficiency and cultural identification will be predicted by *both* heritage language and English usage in informal contexts (i.e., home, friends) *and* more formal individual contexts (i.e., language tapes, language labs, and self-instruction). Altogether, the present study provides a deeper understanding of the interactivity between language and culture in heritage bilinguals.

## Materials and methods

### Participants

Participants included 133 heritage speakers who acquired a non-English native language first and English second, and who rated English as more proficient than their native language on the *Language Experience and Proficiency Questionnaire* (LEAP-Q; Marian et al., 2007). Data were compiled from previous studies conducted in our lab between 2011 and 2022 (i.e., secondary data analysis; Bartolotti et al., 2011; Chabal et al., 2015, 2022; Freeman et al., 2016, 2022; Shook and Marian, 2016; Chen et al., 2017; Marian et al., 2018, 2021; Hayakawa et al., 2020). Participants’ mean age at the time of testing was 23.97 (*SD* = 6.24), and 67% were female. Seventy-nine participants had Spanish as their heritage language, while the remaining 54 participants had a non-Spanish language as their heritage language. The non-Spanish languages all utilized a different script than English and included Korean (*n* = 31), Chinese (*n* = 16), Thai (*n* = 4), Hebrew, Russian, and Tamil (*n* = 1 each). Spanish and non-Spanish heritage speakers did not significantly differ in age (*M* = 24.68 and 22.93, respectively), gender (67.1 and 67.6% female), or years of education (*M* = 14.66 and 15.21), *ps* > 0.137. Non-Spanish bilinguals knew marginally more languages (*M* = 2.43) than Spanish bilinguals (*M* = 2.22), *p* = 0.067. Refer to Table 1 for the linguistic profiles of each group of heritage bilinguals (Spanish and non-Spanish bilinguals), including self-reported heritage language (HL) and English proficiency, ages of HL and English acquisition, and contexts of HL and English acquisition and exposure. Participants had no history of a language or learning disability and had normal or corrected-to-normal vision.

### Materials

#### Language experience and proficiency questionnaire

The *Language Experience and Proficiency Questionnaire* (LEAP-Q; Marian et al., 2007) was used to acquire each participant’s linguistic profile. Participants were asked to list the languages they know in order of dominance as well as acquisition. Information about each language’s (1) acquisition, (2) proficiency, and (3) exposure were obtained. For age of acquisition, participants provided the ages at which they began acquiring, became fluent, began reading, and became fluent at reading each language. For proficiency, participants rated their proficiency in each language in terms of speaking, understanding, and reading on a scale from 0 (None) to 10 (Perfect). For manner of acquisition, participants rated the extent to which various factors contributed to learning each language on a scale from 0 (Not a Contributor) to 10 (Most Important Contributor). These factors included friends, family, reading, language tapes/self-instruction, watching TV, and listening to radio/music. For language exposure, participants rated the extent to which they were currently exposed to each language in various contexts, including friends, family, watching TV, listening to radio/music, reading, and language lab/self-instruction on a scale from 0 (Never) to 10 (Always).

Cultural identification information was obtained by asking participants to list the cultures they identified with and rate the extent to which they identified with each culture on a scale from 0 (No Identification) to 10 (Complete Identification). Cultural identification with the HL and English was determined based on ratings given to cultures associated with each language (e.g., “Korea” for cultural identification with Korean as a HL, “USA” for cultural identification with English). If more than one culture associated with a language was listed (e.g., Culture 1: “Latino” and Culture 2: “Mexican” for Spanish), we selected the rating for the culture that was ranked highest. In addition to linguistic and cultural information, demographic information such as age, gender, years of formal education, highest level of education, year of migration to the United States (if applicable), and any history of vision, hearing, language, or learning disabilities were provided by each participant. Although some participants were fluent in a third language, we did not analyze the third language information due to the small number of participants who were fluent in a third language.

### Procedure

All studies included in the secondary analysis were reviewed and approved by Northwestern University’s Institutional Review Board. In all studies, the *Language Experience and Proficiency Questionnaire* (Marian et al., 2007) was administered toward the end of the testing session. Participants provided informed consent prior to the start of the experiment and were debriefed at the end of the experiment.

### Data analysis

Two sets of analyses were conducted to examine predictors of heritage language (HL) and English proficiency and cultural identification among Spanish and non-Spanish heritage speakers. To address issues of multicollinearity, we began by examining the correlational structure of LEAP-Q measures and created 7 composite measures for each language, which included **Age of Acquisition** (AoA), **Duration of Immersion** (average number of years immersed in a country, school, or workplace in which each language was spoken), and five composite measures which each represented an aggregated measure of manner of acquisition and current exposure in different contexts. The included contexts were **Family Acquisition and Exposure** (averaged across ratings of how much family contributed to the acquisition of each language and how much participants are currently exposed to each language through family), **Friends Acquisition and Exposure**, **Media Acquisition and Exposure** (e.g., through TV, radio), **Reading Acquisition and Exposure**, and **Individual Acquisition and Exposure** (e.g., through language tapes/language lab/self-instruction). In order to assess the impact of relative language experience, we additionally calculated a dominance score for each composite measure by subtracting the HL score from the English score. All fixed effects had VIF scores < 5, indicating minimal multicollinearity.

Effects of HL, English, and relative language experience measures within each set of analyses were examined with separate linear mixed-effects models, with variable numbers of participants depending on the availability of relevant proficiency or cultural identification measures for individual subjects. Models therefore included effects of (1) HL experience on HL and English receptive (averaged across understanding and reading) and expressive (speaking) proficiency (*n* = 126), (2) English experience on HL and English receptive and expressive proficiency (*n* = 127), (3) relative language experience on HL and English receptive and expressive proficiency (*n* = 126), (4) HL experience on HL and English cultural identification (*n* = 79), (5) English experience on HL and English cultural identification (*n* = 79), and (6) relative language experience on HL and English cultural identification (*n* = 79).

Fixed effects for proficiency models included the 7 HL, English, or relative language experience composite measures plus all two-, three-, and four-way interactions with Heritage Group (Spanish vs. non-Spanish), Language (HL vs. English proficiency), and Measure (receptive vs. expressive proficiency). Cultural identification models included the 7 composite measures, receptive and expressive proficiency, plus all two- and three-way interactions with Heritage Group (Spanish vs. non-Spanish) and Language (HL vs. English cultural identification). All models included a random intercept for participant. Contrasts for Heritage Group (Spanish: −0.57 vs. Non-Spanish: +0.43), Language (HL: −0.5 vs. English: +0.5), and Measure (Expressive: −0.5 vs. Receptive: +0.5) were centered and weighted by the number of responses. Continuous fixed effects were mean-centered and scaled *via* z-score transformation.

Parameter estimates and significance of fixed effects were assessed with the Satterwhite method using the *lme4* (Bates et al., 2014) and *lmerTest* (Kuznetsova et al., 2017) R packages. Tukey-adjusted follow-up tests of simple effects were conducted using the *emmeans* and *emtrends* functions of the *emmeans* R package (Lenth et al., 2018).

## Results

### Predictors of heritage language and English proficiency

#### Effects of heritage language (HL) experience

Self-reported proficiency was significantly higher in English (*M* = 9.55, 95% CI [9.38, 9.73]) than in the heritage language (*M* = 7.93, 95% CI [7.76, 8.10]), *p* <0.001. See Table 2 for full output. A two-way interaction between Language and Heritage Group (*p* = 0.014) indicated that Spanish bilinguals had significantly higher HL proficiency than non-Spanish bilinguals [*Estimate* = 0.64, *SE* = 0.17, *t*_(163.94)_ = 3.70, *p* < 0.001], whereas the two groups did not differ in English proficiency [*Estimate* = 0.27, *SE* = 0.17, *t*_(163.94)_ = 1.53, *p* = 0.128].

**FIGURE 1:**
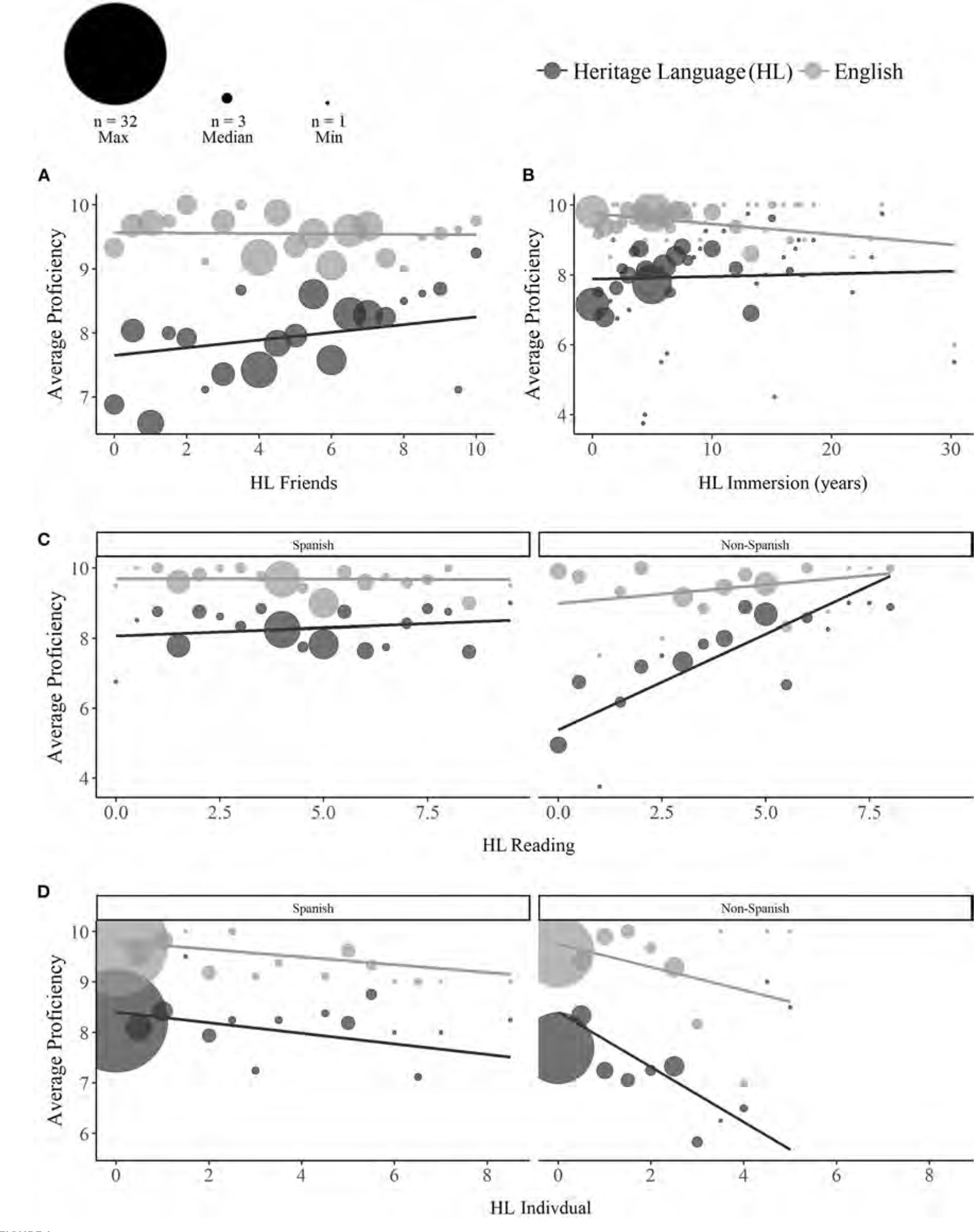
Effects of heritage language (HL) experience on self-reported HL (dark gray) and English (light gray) proficiency. Across both groups, HL proficiency increased with greater HL acquisition and exposure through friends **(A)**, while English proficiency decreased with greater HL immersion duration **(B)**. HL proficiency increased with greater HL reading acquisition and exposure for non-Spanish, but not Spanish bilinguals **(C)**. Overall proficiency decreased with greater HL individual acquisition and exposure, which was particularly the case for HL proficiency among non-Spanish bilinguals **(D)**. Dot sizes reflect the number of participants contributing to each aggregated value (max = 32, median = 3, min = 1).

Across both groups, the composite measures of HL Acquisition and Exposure through **friends** (Language x Friends: *p* = 0.023; Figure 1A) and **reading** (Language x Reading: *p* < 0.001) predicted higher self-reported HL proficiency [Friends: *Estimate* = 0.16, *SE* = 0.09, *t*_(163.94)_ = 1.81, *p* = 0.070; Reading: *Estimate* = 0.68, *SE* = 0.09, *t*_(163.94)_ = 7.33, *p* < 0.001], but not English proficiency (*ps* > 0.202). The effect of reading experience was greater for receptive HL proficiency [*Estimate* = 0.89, *SE* = 0.11, *t*_(274)_ = 8.18, *p* < 0.001] compared to expressive proficiency [*Estimate* = 0.47, *SE* = 0.11, *t*_(274)_ = 4.27, *p* < 0.001; Language x Measure x Reading: *p* = 0.017].

In contrast, a two-way interaction between Language and Immersion (*p* = 0.002) revealed that a longer duration of HL **immersion** was associated with significantly lower English proficiency [*Estimate* =−0.17, *SE* = 0.08, *t*_(163.94)_ =−2.03, *p* = 0.044], but not HL proficiency [*Estimate* = 0.05, *SE* = 0.08, *t*_(163.94)_ = 0.53, *p* = 0.595; Figure 1B]. Greater HL acquisition and exposure through **individual** contexts (e.g., self-instruction, language labs, and language tapes) was associated with lower proficiency overall (*p* < 0.001), which was particularly the case for HL proficiency [*Estimate* = −0.63, *SE* = 0.10, *t*_(164)_ =−6.25, *p* < 0.001] compared to English proficiency [*Estimate* =−0.30, *SE* = 0.10, *t*_(164)_ =−2.91, *p* = 0.004; Language x Individual: *p* = 0.001].

Finally, three-way interactions with Language and Heritage Group revealed that ratings of HL acquisition and exposure through **reading** (*p* < 0.001) and **individual** contexts (*p* = 0.002) were more predictive of HL proficiency for non-Spanish bilinguals [Reading: *Estimate* = 1.25, *SE* = 0.15, *t*_(164)_ = 8.57, *p* < 0.001; Individual: *Estimate* = −1.06, *SE* = 0.17, *t*_(164)_ =−6.36, *p* < 0.001] than Spanish bilinguals [Reading: *Estimate* = 0.25, *SE* = 0.15, *t*_(164)_ = 1.68, *p* = 0.095; Individual: *Estimate* =−0.21, *SE* = 0.12, *t*_(164)_ =−1.78, *p* = 0.077; see Figures 1C,D].

#### Effects of English experience

Earlier **ages of English acquisition** (Language x AoA: *p* < 0.001) and higher ratings of English acquisition and exposure through **family** (Language x Family: *p* = 0.012) predicted lower HL proficiency [AoA: *Estimate* = 0.36, *SE* = 0.10, *t*_(163)_ = 3.50, *p* < 0.001; Family: *Estimate* =−0.34, *SE* = 0.11, *t*_(163)_ =−3.04, *p* < 0.001], but not English proficiency (*ps* > 0.48). Refer to Table 3 for full output. Although simple effects did not reach significance, an interaction between Language and Reading (*p* = 0.016) indicated that higher ratings of English acquisition and exposure through **reading** were associated with lower HL proficiency [*Estimate* =−0.08, *SE* = 0.12, *t*_(163)_ =−0.62, *p* = 0.536], but higher English proficiency [*Estimate* = 0.18, *SE* = 0.12, *t*_(163)_ = 1.52, *p* = 0.131].

**FIGURE 2:**
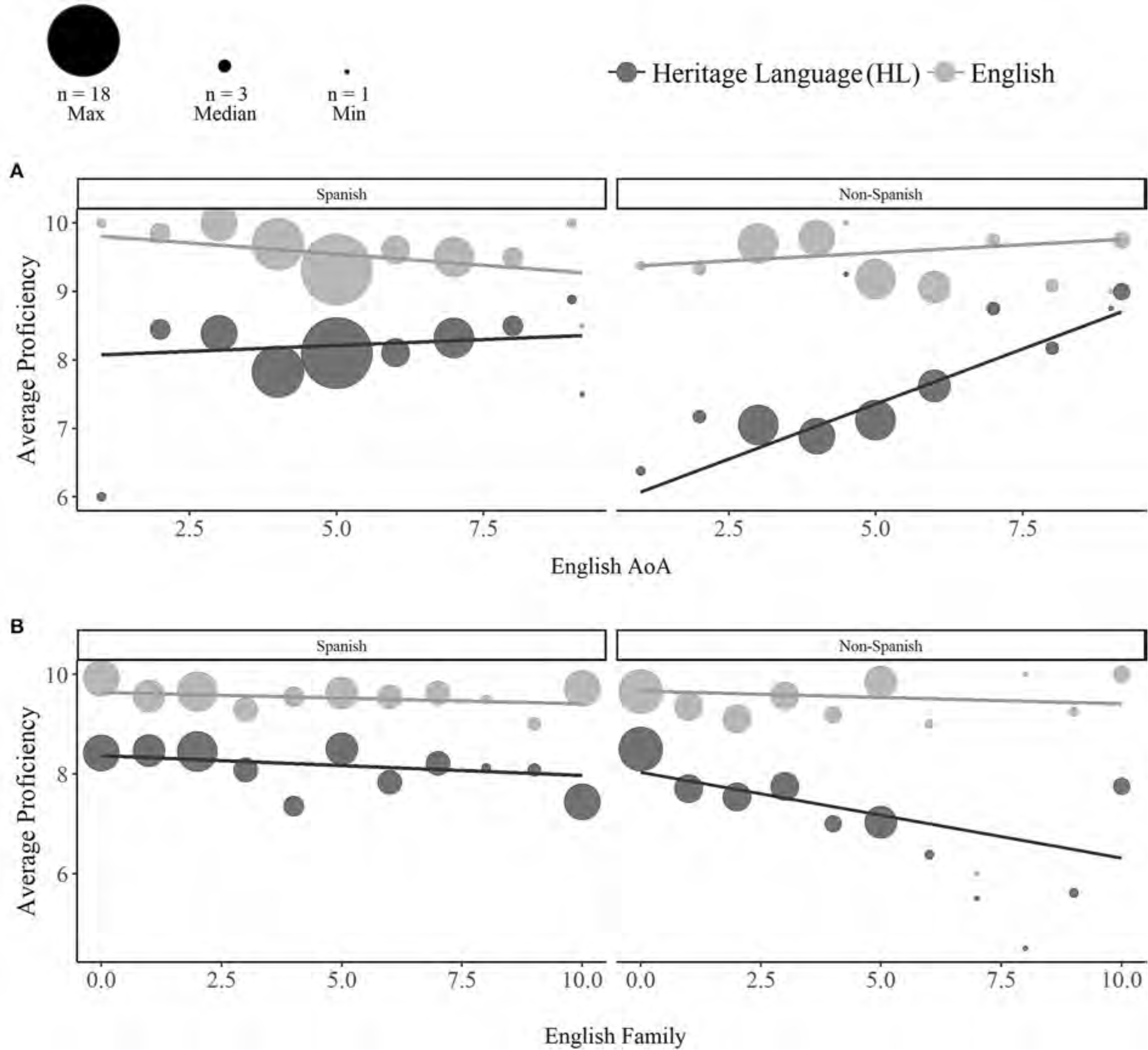
Effects of English experience on self-reported HL (dark gray) and English (light gray) proficiency. Among non-Spanish bilinguals, HL proficiency increased with later ages of English acquisition **(A)** and decreased with greater English acquisition and exposure through family **(B)**. Dot sizes reflect the number of participants contributing to each aggregated value (max = 18, median = 3, min = 1).

Three-way interactions with Language and Heritage Group revealed that the effects of **age of English acquisition** (*p* = 0.046) and **family** (*p* = 0.029) on HL proficiency were greater for non-Spanish bilinguals [AoA: *Estimate* = 0.65, *SE* = 0.15, *t*_(163)_ = 4.30, *p* < 0.001; Family: *Estimate* =−0.55, *SE* = 0.19, *t*_(163)_ =−2.97, *p* = 0.003] than Spanish bilinguals [AoA: *Estimate* = 0.07, *SE* = 0.13, *t*_(163)_ = 0.50, *p* = 0.62; Family: *Estimate* =−0.13, *SE* = 0.12, *t*_(163)_ =−1.02, *p* = 0.311; see Figures 2A,B, respectively].

#### Effects of relative language experience (English—HL)

**FIGURE 3:**
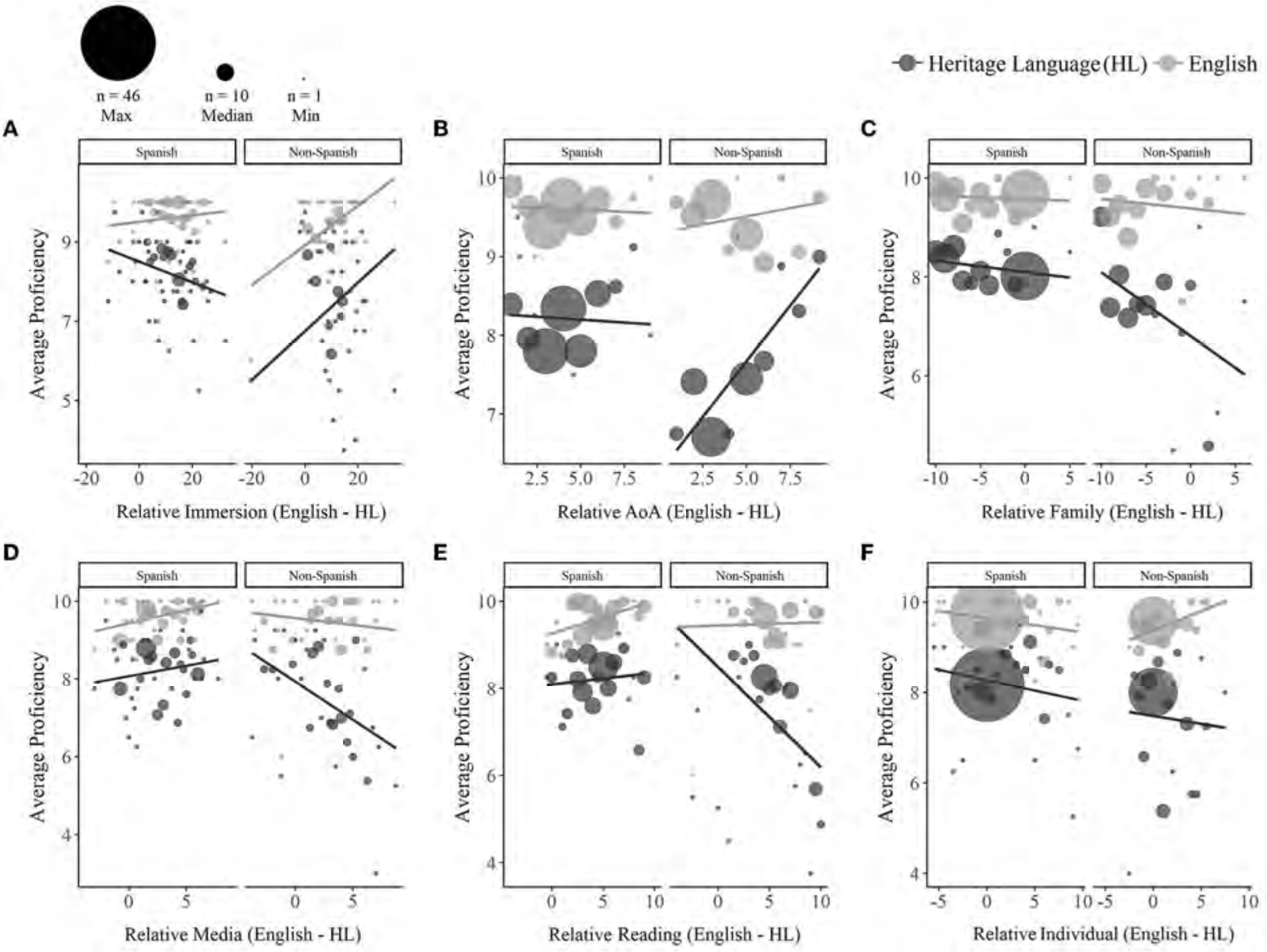
Effects of relative language experience (English—HL) on self-reported HL (dark gray) and English (light gray) proficiency. Among non-Spanish bilinguals, both HL and English proficiency increased with relatively greater English (vs. HL) immersion, while for Spanish bilinguals, HL proficiency decreased with relatively greater English immersion **(A)**. Among non-Spanish bilinguals, lower HL proficiency was predicted by relatively earlier ages of English (vs. HL) acquisition **(B)**, as well as relatively higher ratings of English (vs. HL) acquisition and exposure through family **(C)**, media **(D)**, and reading **(E)**. Higher ratings of English (vs. HL) acquisition and exposure in individual contexts was non-significantly associated with higher English proficiency and lower HL proficiency **(F)**. Dot sizes reflect the number of participants contributing to each aggregated value (max = 46, median = 10, min = 1).

A significant three-way interaction between Language, Heritage Group, and **relative immersion** (*p* = 0.016) revealed that among non-Spanish bilinguals, relatively longer English (vs. HL) immersion predicted higher self-reported proficiency in both the HL [*Estimate* = 0.53, *SE* = 0.17, *t*_(151)_ = 3.16, *p* = 0.002] and in English [*Estimate* = 0.43, *SE* = 0.17, *t*_(151)_ = 2.58, *p* = 0.011]. Among Spanish bilinguals, relatively longer English immersion was associated with marginally lower HL proficiency [*Estimate* =−0.22, *SE* = 0.12, *t*_(151)_ =−1.90, *p* = 0.059], with no effect on English proficiency (*p* = 0.549; see Figure 3A and Table 4 for full output).

A series of three-way interactions additionally emerged for **relative age of acquisition** (*p* < 0.001) and the composite measures for relative acquisition and exposure through **family** (*p* = 0.007), **media** (*p* = 0.008), and **reading** (*p* < 0.001). Among non-Spanish bilinguals, HL proficiency was negatively predicted by more similar ages of HL and English acquisition [*Estimate* = 0.60, *SE* = 0.14, *t*_(151)_ = 4.41, *p* < 0.001] and relatively higher ratings of English (vs. HL) acquisition and exposure experience through family [*Estimate* =−0.50, *SE* = 0.13, *t*_(151)_ =−3.85, *p* < 0.002], media [*Estimate* = −0.52, *SE* = 0.14, *t*_(151)_ = −3.76, *p* < 0.002], and reading [*Estimate* = −0.63, *SE* = 0.13, *t*_(151)_ = −4.82, *p* < 0.001]. Relative AoA, manner of acquisition, and exposure did not predict HL proficiency for Spanish bilinguals (*ps* > 0.242) or English proficiency for either group (*ps* > 0.118). Finally, a significant two-way interaction between Language and relative individual experience [*Estimate* = 0.17, *SE* = 0.07, *t*_(327)_ = 2.34, *p* = 0.020] indicated that greater English (vs. HL) acquisition and exposure in individual contexts was (non-significantly) associated with lower HL proficiency [*Estimate* = −0.11, *SE* = 0.10, *t*_(151)_ =−1.15, *p* = 0.252] and higher English proficiency [*Estimate* = 0.08, *SE* = 0.10, *t*_(151)_ = 0.79, *p* = 0.438; see Figures 3B–F].

**FIGURE 4:**
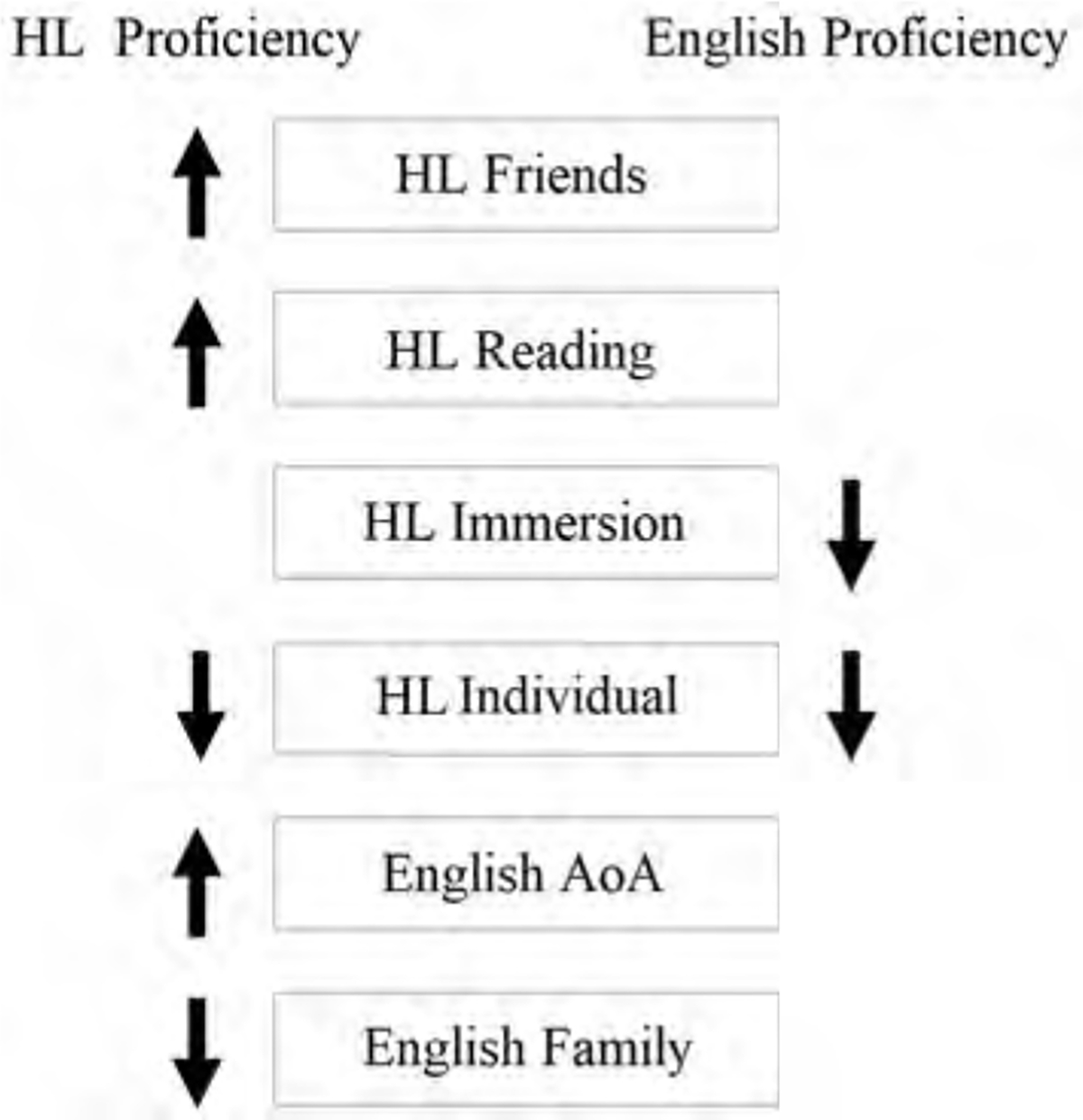
Overview of heritage language (HL) and English experience effects on self-reported HL and English proficiency.

In sum, higher HL proficiency was predicted by greater HL experience through reading and friends, later absolute and relative ages of English acquisition, less absolute and relative English experience through family, and less relative English experience through reading and media. HL reading experience had a greater impact on HL receptive proficiency (understanding/reading) compared to expressive proficiency (speaking). English proficiency declined with longer durations of HL immersion, and proficiency in both languages increased with longer relative durations of English (vs. HL) language immersion. Proficiency in both languages declined with greater HL experience in individual contexts (see Figure 4). Notably, effects of both HL and English experience were generally more robust among non-Spanish compared to Spanish bilinguals.

### Predictors of heritage language and English cultural identification

#### Effects of heritage language experience

Cultural identification was significantly higher with the heritage language (*M* = 7.71, 95% CI [7.07, 8.35]) than English (*M* = 6.74, 95% CI [6.09, 7.38]), *p* = 0.035. See Table 5 for full output.

**FIGURE 5:**
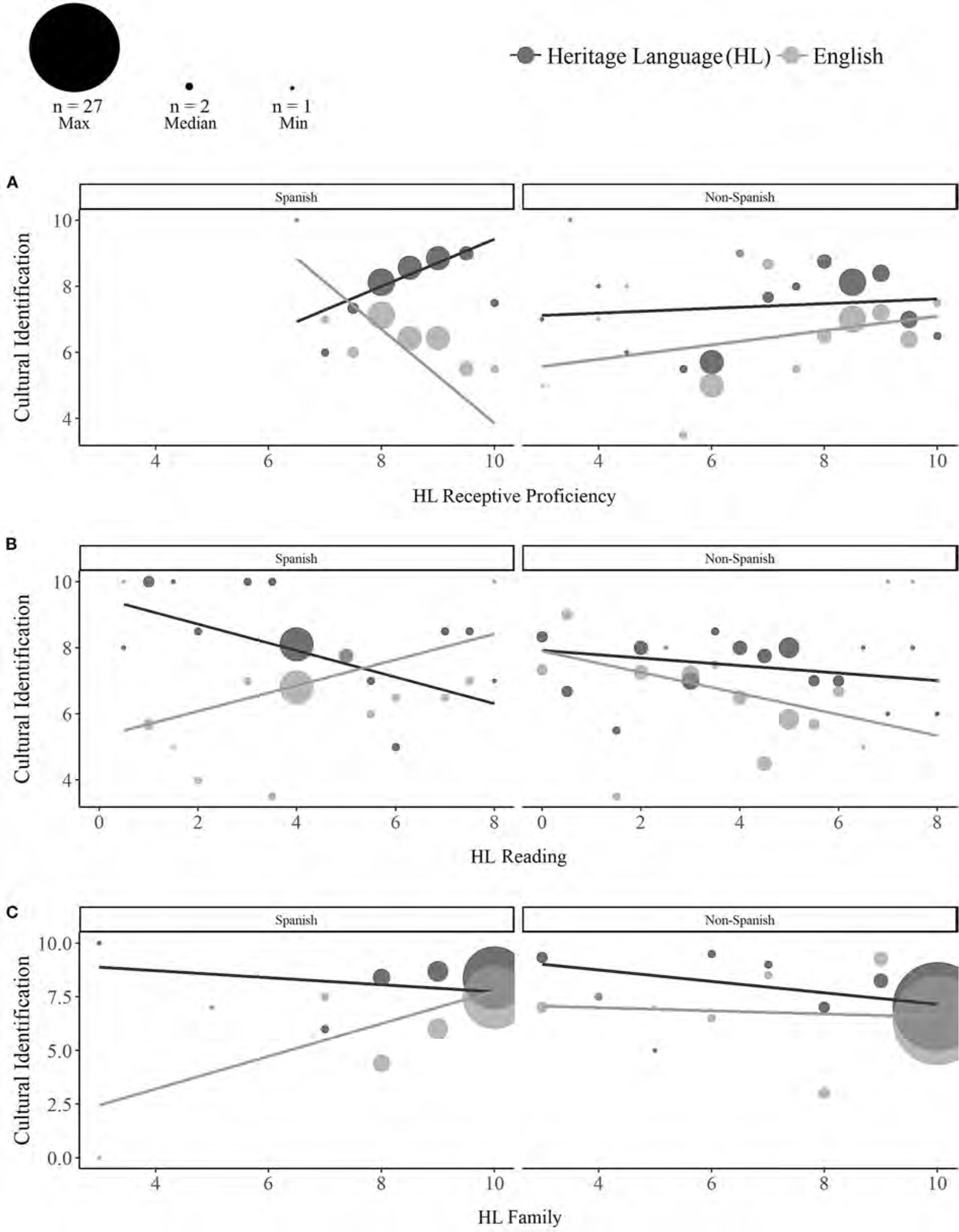
Effects of heritage language (HL) experience on cultural identification with the HL (dark gray) and English (light gray). Among Spanish bilinguals, cultural identification with English increased with lower HL receptive proficiency **(A)**, as well as greater HL acquisition and exposure through reading **(B)** and family **(C)**. Dot sizes reflect the number of participants contributing to each aggregated value (max = 27, median = 2, min = 1).

A three-way interaction between Language, Heritage Group, and self-reported Receptive Proficiency (*p* = 0.020) revealed that among Spanish bilinguals, cultural identification with English (but not the HL) declined with higher **receptive proficiency** in the HL [*Estimate* =−2.16, *SE* = 0.89, *t*_(116)_ =−2.43, *p* = 0.017; Figure 5A]. A three-way interaction with Reading (*p* = 0.042) revealed that among Spanish bilinguals, higher ratings of HL acquisition and exposure through **reading** [*Estimate* = 0.79, *SE* = 0.43, *t*_(116)_ = 1.83, *p* = 0.062] were associated with greater cultural identification with English, but lower cultural identification with the HL [*Estimate* = −0.82, *SE* = 0.43, *t*_(118)_ =−1.89, *p* = 0.071; Figure 5B]. Cultural identification with English was not moderated by receptive HL proficiency (*p* = 0.834) or reading experience (*p* = 0.669) among non-Spanish bilinguals.

Finally, the composite measure of **family** HL acquisition and exposure was unexpectedly associated with increased cultural identification with English [*Estimate* = 0.69, *SE* = 0.33, *t*_(116)_ = 2.08, *p* = 0.040], but not the HL (*p* = 0.195; Language × Family: *p* = 0.023). Although the three-way interaction with Heritage Group was not significant (*p* = 0.123), simple effects revealed that the effect of HL family experience on English identification was driven by Spanish [*Estimate* = 1.52, *SE* = 0.57, *t*_(116)_ = 2.65, *p* = 0.009] rather than non-Spanish bilinguals [*Estimate* =−0.15, *SE* = 0.46, *t*_(116)_ =−0.45, *p* = 0.65; see Figure 5C]. No significant effects of English experience were observed for cultural identification (see Supplementary Table 1 for full output).

#### Effects of relative language experience (English—HL)

**FIGURE 6:**
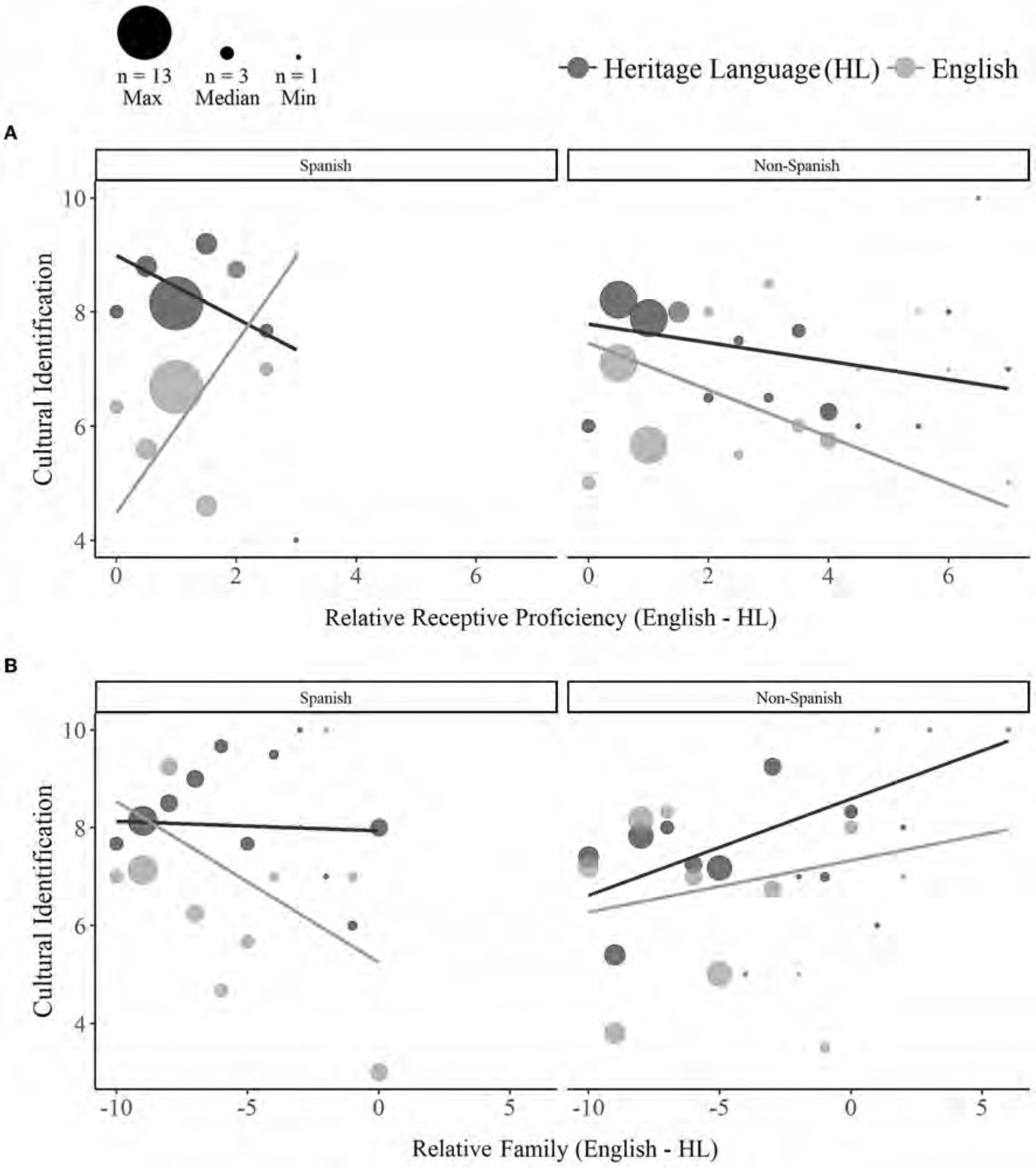
Effects of relative language experience (English—HL) on cultural identification with the HL (dark gray) and English (light gray). Among Spanish bilinguals, cultural identification with English increased with higher relative English (vs. HL) receptive proficiency **(A)**. Relatively higher ratings of English (vs. HL) acquisition and exposure through family was associated with lower English identification among Spanish bilinguals and marginally higher HL identification among non-Spanish bilinguals **(B)**. Dot sizes reflect the number of participants contributing to each aggregated value (max = 13, median = 3, min = 1).

A significant main effect of relative **immersion** indicated that relatively longer durations of English (vs. HL) immersion predicted greater cultural identification with both languages (*p* = 0.019; see Table 6 for full output). Consistent with the effect of self-reported HL receptive proficiency, a three-way interaction between Language, Heritage Group, and relative self-reported **receptive proficiency** (*p* = 0.041) indicated that among Spanish bilinguals, cultural identification with English (but not the HL) increased with greater relative English (vs. HL) receptive proficiency [*Estimate* = 2.28, *SE* = 1.04, *t*_(116)_ = 2.18, *p* = 0.031; see Figure 6A]. Relative proficiency did not moderate cultural identification with either language for non-Spanish bilinguals (*ps* > 0.42). A significant interaction between Heritage Group and relative **family** acquisition and exposure (*p* = 0.021) indicated that relative English (vs. HL) family experience was a (non-significant) negative predictor of overall cultural identification among Spanish bilinguals [*Estimate* =−0.64, *SE* = 0.42, *t*_(58)_ =−1.52, *p* = 0.135] and a marginally positive predictor of overall identification among non-Spanish bilinguals [*Estimate* = 0.56, *SE* = 0.29, *t*_(58)_ = 1.93, *p* = 0.058]. Although the three-way interaction with Language did not approach significance (*p* = 0.435), relatively greater English (vs. HL) family experience was associated with significantly lower cultural identification with English among Spanish bilinguals [*Estimate* =−1.21, *SE* = 0.6, *t*_(116)_ =−2.03, *p* = 0.045] and marginally greater cultural identification with the HL among non-Spanish bilinguals [*Estimate* = 0.73, *SE* = 0.41, *t*_(116)_ = 1.78, *p* = 0.08; see Figure 6B].

## Discussion

The goal of the present study was to uncover linguistic predictors of self-reported language proficiency and cultural identification among different groups of adult heritage bilinguals. Self-reported proficiency in the majority language (English) was best predicted by the duration of immersion in the heritage language (HL). As expected, a longer cumulative duration of immersion in a country or school and/or work environment in which the HL was spoken was associated with lower reported English proficiency. Higher reported HL proficiency was predicted by higher ratings of HL acquisition and use through reading and friends, lower ratings of English acquisition and use through family, and later ages of English acquisition. Proficiency in both languages declined with greater HL experience in individual contexts (e.g., acquisition and exposure through self-instruction, language tapes, and language labs). Finally, despite higher self-reported English proficiency, cultural identification was higher with the HL, and this was especially true for Spanish heritage bilinguals. English cultural identification was negatively associated with subjective HL receptive proficiency, and to a lesser extent, positively associated with greater reliance on reading and family for HL acquisition and use. In addition to characterizing the factors that promote language proficiency and cultural identification, a critical finding from the present investigation is that the impact of heritage language and English language experience varied depending on heritage speakers’ native languages.

### Heritage group and self-reported language proficiency

First, we found that greater reliance on reading for HL acquisition and exposure predicted higher self-reported HL receptive proficiency among non-Spanish, but not Spanish bilinguals. One probable explanation for this finding is that the two groups differed in how much they could rely on English reading skills to support literacy in the HL. Unlike Spanish-English bilinguals, the non-Spanish bilinguals’ heritage languages (Chinese, Hebrew, Korean, Russian, Tamil, and Thai) all utilized a different script from English, which may have reduced the amount of cross-linguistic transfer in literacy (Huang and Hanley, 1995; Durgunoglu, 2002; Lindsey et al., 2003; Bialystok et al., 2005a,b; Luk, 2005) and other academic skills (Van der Slik, 2010; Zhang, 2013; Kostelecká et al., 2015; Siu and Ho, 2015; see Koda, 2005; Genesee et al., 2006 for reviews). For instance, Bialystok et al. (2005a) observed that same-script bilinguals transferred literacy skills across languages, while different-script bilinguals did not. Because Spanish and English utilize the same script, the ability to comprehend written text in Spanish may be supported by English reading skills even without extensive exposure to Spanish text. In contrast, for different-script bilinguals, the ability to comprehend written text in the heritage language may be more contingent on dedicated exposure to HL text through reading. Consistent with this interpretation, non-Spanish bilinguals with minimal HL reading experience had significantly lower reading proficiency than matched Spanish bilinguals (−1 SD; *Ms* = 4.33 and 7.30, respectively; *p* < 0.001). This gap closed among non-Spanish and Spanish bilinguals with greater HL reading experience (+1 SD; *Ms* = 8.67 and 7.80, respectively; *p* = 0.070). This finding suggests that HL experience through reading may be particularly important for different-script bilinguals.

We additionally found that earlier ages of English acquisition and a more substantial role of family for English acquisition and use predicted lower self-reported HL proficiency among non-Spanish, but not Spanish bilinguals. Because bilinguals need to split their time between their two languages, time spent using one language leads to decreased use of the other language (Meir and Janssen, 2021). HL proficiency often declines with greater majority language use and less HL use (Jia and Aaronson, 2003; Gollan et al., 2015; Montrul, 2016; Vorobyeva and Bel, 2021). Our findings suggest that the negative impact of reduced HL use on HL proficiency may be minimized for speakers of more typologically similar languages, potentially because reading and conversational skills acquired from the majority language can transfer to the HL. In addition to orthographic similarities, the degree of lexical and grammatical overlap between English and Spanish (two Indo-European languages) is likely greater than between English and non-Spanish languages (primarily non-Indo-European) included in the present study. Consequently, even if time spent using English detracts from time spent using the HL, Spanish-English bilinguals may be better able to benefit from positive linguistic transfer between languages (Odlin, 1989; Bialystok et al., 2003, 2005a; Melby-Lervåg and Lervåg, 2011).

Due to the high number of Spanish speakers in the United States, Spanish-English bilinguals may also be able to benefit from greater HL experience outside of the home even if English is used more frequently with family. Indeed, Spanish bilinguals in the present study did report significantly greater HL exposure through music/radio, reading, and individual instruction, as well as numerically greater HL exposure through family and TV relative to non-Spanish bilinguals (see Table 1). Supplementary analyses provide preliminary support for such a compensatory mechanism, as the negative effects of both English AoA and family use on HL proficiency declined with greater overall HL experience (aggregated across contexts of acquisition and exposure; see Supplementary Table 2 for details). Together, these findings suggest that the extent to which majority language experience helps vs. hinders HL acquisition and maintenance is subject to variability in linguistic similarity across languages, as well as the amount of HL use across different contexts.

### Heritage group and cultural identification

Among Spanish bilinguals, identification with English-speaking cultures (e.g., American) increased with lower self-reported HL receptive proficiency, as well as with greater HL experience through reading and family. Prior work has demonstrated a robust relationship between cultural identification and language proficiency, most often showing a positive association between cultural identification and proficiency within a given language (e.g., between HL proficiency and HL ethnic identity; Bankston and Zhou, 1995; Cho, 2000; Pease-Alvarez, 2002; Oh and Fuligni, 2010; Yu, 2015; Arredondo et al., 2016; Schroeder et al., 2017). Our findings indicate that proficiency in one language can be inversely related to cultural identification with the other, and that the relationship between language experience and cultural identification varies across different groups of heritage speakers.

More unexpected was our finding that cultural identification with English increased with higher ratings of Spanish acquisition and exposure through reading and family. Effects of relative proficiency further indicated that while identification with the two languages was comparably high among Spanish bilinguals with substantially greater Spanish (vs. English) family experience, identification with English became progressively lower with more balanced use of the two languages at home. Although speculative, some heritage speakers may develop stronger or weaker identification with each culture to compensate for imbalances in language use and immersion at home. Cheryan and Monin (2005) found that Asian Americans expressed greater American cultural identification when their American identities were threatened. Additionally, while bilinguals primed with a particular language or culture often exhibit culturally-congruent behaviors and judgments (i.e., assimilation), there are also cases in which bilinguals instead respond in culturally-incongruent ways, particularly if they perceive their cultural identities to be threatened or in conflict with one another (Benet-Martínez et al., 2002; Cheng et al., 2006; Zou et al., 2008). The fact that a positive association between English identification and HL experience was found for Spanish bilinguals, but not for the non-Spanish bilinguals may potentially stem from differences in the extent to which the two groups perceive their cultural identities to be compatible vs. in conflict. Indeed, an exploratory examination of the relationship between English and HL identification within the two groups provides tentative support for this interpretation. Specifically, while there was a non-significant negative correlation between English and HL cultural identification among Spanish bilinguals (*r* =−0.22, *p* = 0.206), there was a marginal positive correlation between identification with the two languages among non-Spanish bilinguals (*r* = 0.29, *p* = 0.059). Similar group differences were observed by Gong (2007) who found that while there was no correlation between ethnic (minority culture) identity and national (majority culture) identity among African Americans, ethnic and national identity were positively correlated among American-born Chinese Americans. A possible avenue for future research may therefore be to examine whether different groups of heritage speakers vary in the perceived compatibility of their two cultures, and whether such differences moderate the impact of language experience on cultural identification within and across languages.

Future research may additionally examine the extent to which the observed effects and predictors of self-reported language proficiency are replicated using objective measures of language ability. The inclusion of objective measures assessing a variety of linguistic domains (e.g., lexicon, syntax, pronunciation) will contribute to determining the generalizability of the present findings and for characterizing the impact of heritage and majority language experience on different aspects of language proficiency. Second, our understanding of systematic variability across different heritage speakers would benefit from the inclusion of a greater number of participants from a more diverse range of language backgrounds. In particular, the roles of script and cross-linguistic transfer could be more fully elucidated through the inclusion of same- and different-script bilinguals within (e.g., German and Italian vs. German and Greek) and across (e.g., German and Vietnamese vs. German and Mandarin) language families. Likewise, interactions between language experience and culture could be examined more fully by crossing linguistic and cultural similarity.

In conclusion, the present findings reveal that the relationships between language experience, self-reported language proficiency, and cultural identification systematically vary as a function of heritage speakers’ native languages. We additionally provide preliminary evidence to suggest that such differences may partly stem from variability in the degree of linguistic (e.g., orthographic overlap) and cultural (e.g., cultural compatibility) similarity across languages, as well as in opportunities for HL exposure outside of the home. Together, our results demonstrate the complex interplay between heritage and majority language experience, and highlight the need to consider individual measures within the broader context of bilinguals’ linguistic environments and history. Greater sensitivity to the needs and abilities of different types of bilinguals can promote the development of more effective heritage bilingual curricula, and provide a more nuanced understanding of heritage bilinguals’ language acquisition and identity.

## Supplementary Material

Supplementary materials

## Figures and Tables

**TABLE 1:** Linguistic profiles of Spanish and non-Spanish heritage speakers.

Measure	Heritage group	Heritage language	English	HL vs. English

***Proficiency (0 = None to 10 = Perfect)***				
Speaking	Spanish	8.08 (1.10)	9.56 (0.66)	***
	Non-Spanish	7.72 (1.60)	9.43 (0.79)	***
Reading	Spanish	7.591.526.042.89	9.65 (0.60)	***
	Non-Spanish		9.44 (0.86)	***
Understanding	Spanish	8.76 (1.05)	9.68 (0.63)	***
	Non-Spanish	8.43 (1.27)	9.52 (0.84)	***
***Age of Acquisition***				
Overall Acquisition	Spanish	1.091.140.480.67	4.93 (1.89)	***
	Non-Spanish		4.95 (2.13)	***
Reading Acquisition	Spanish	5.90 (2.54)	6.02 (1.77)	
	Non-Spanish	4.97 (2.85)	6.04 (2.18)	*
***Context of Acquisition (0 = Not a Contributor to 10 = Most Important Contributor)***				
Family	Spanish	9.38 (1.75)	4.04 (3.35)	***
	Non-Spanish	9.46 (0.95)	3.41 (3.20)	***
Friends	Spanish	5.15 (3.20)	8.66 (1.82)	***
	Non-Spanish	5.56 (3.15)	9.04 (1.72)	***
Individual (Language Tapes/Self instruction)	Spanish	1.37 (2.18)	3.34 (3.91)	***
	Non-Spanish	1.69 (2.25)	2.80 (3.36)	*
TV	Spanish	5.95 (2.98)	8.151.796.432.48	***
	Non-Spanish	5.76 (2.96)		
Radio/Music	Spanish	5.723.232.092.61	6.953.073.983.32	**
	Non-Spanish			***
Reading	Spanish	5.72 (2.88)	8.86 (1.46)	***
	Non-Spanish	4.91 (2.99)	8.37 (2.56)	***
***Context of Exposure (0 = Never to 10 = Always)***				
Family	Spanish	8.93 (2.1)	4.40 (3.29)	***
	Non-Spanish	8.48 (2.3)	3.39 (3.04)	***
Friends	Spanish	3.81 (2.90)	8.81 (2.00)	***
	Non-Spanish	3.94 (2.92)	8.98 (1.93)	***
Individual (Language-Lab/Self-instruction)	Spanish	2.010.722.881.37	2.923.761.653.11	*
	Non-Spanish			**
TV	Spanish	4.18 (3.02)	8.701.777.173.01	***
	Non-Spanish	3.65 (3.27)		***
Radio/Music	Spanish	5.303.383.943.19	7.93 (2.13)	***
	Non-Spanish		7.48 (2.56)	***
Reading	Spanish	3.402.372.172.20	8.73 (1.76)	***
	Non-Spanish		8.44 (2.82)	***
***Immersion (years)***				
Family	Spanish	23.578.3420.856.77	15.8611.389.4011.17	***
	Non-Spanish			***
Country	Spanish	7.53 (8.21)	21.746.4717.286.56	***
	Non-Spanish	6.18 (5.35)		***
School/Work	Spanish	7.92 (7.99)	18.056.2715.695.60	***
	Non-Spanish	5.78 (5.85)		***

Values in parentheses represent standard deviations. Boxed values indicate significant differences between Spanish and non-Spanish heritage speakers (across rows; p < 0.05). Asterisks represent significant differences between the Heritage Language and English for each group (across columns).

* p < 0.05

** p < 0.01

*** p < 0.001.

**TABLE 2:** Effects of heritage language experience on self-reported heritage language and English proficiency.

	*Estimate*	*SE*	*df*	*t*	*p*	

(Intercept)	8.77	0.08	109	116.19	<0.001	***
Language	1.60	0.07	327	21.67	<0.001	***
Heritage group	−0.46	0.16	109	−2.91	0.004	**
Measure	−0.04	0.07	327	−0.57	0.566	
AoA	−0.10	0.08	109	−1.25	0.215	
Immersion	−0.05	0.07	109	−0.65	0.519	
Family	−0.07	0.07	109	−0.97	0.337	
Friends	0.10	0.08	109	1.28	0.205	
Media	0.00	0.08	109	0.02	0.983	
Reading	0.35	0.08	109	4.33	<0.001	***
Individual	−0.42	0.09	109	−4.86	<0.001	***
Language:Heritage	0.38	0.15	327	2.47	0.014	*
Language:Measure	0.30	0.15	327	2.02	0.044	*
Heritage:Measure	−0.31	0.15	327	−2.02	0.044	*
Language:AoA	0.10	0.08	327	1.24	0.217	
Heritage:AoA	0.02	0.18	109	0.09	0.930	
Measure:AoA	−0.07	0.08	327	−0.83	0.410	
Language:Immersion	−0.22	0.07	327	−3.05	0.002	**
Heritage:Immersion	−0.24	0.15	109	−1.56	0.121	
Measure:Immersion	0.04	0.07	327	0.54	0.592	
Language:Family	−0.06	0.07	327	−0.82	0.413	
Heritage:Family	0.03	0.14	109	0.19	0.853	
Measure:Family	−0.11	0.07	327	−1.66	0.098	
Language:Friends	−0.17	0.08	327	−2.29	0.023	*
Heritage:Friends	−0.35	0.16	109	−2.18	0.031	*
Measure:Friends	0.01	0.08	327	0.20	0.844	
Language:Media	−0.05	0.08	327	−0.62	0.533	
Heritage:Media	0.23	0.16	109	1.44	0.153	
Measure:Media	0.01	0.08	327	0.07	0.945	
Language:Reading	−0.50	0.08	327	−6.31	<0.001	***
Heritage:Reading	0.70	0.17	109	4.22	<0.001	***
Measure:Reading	0.20	0.08	327	2.55	0.011	*
Language:Individual	0.30	0.09	327	3.51	0.001	**
Heritage:Individual	−0.57	0.18	109	−3.16	0.002	**
Measure:Individual	−0.06	0.09	327	−0.69	0.488	
Language:Heritage:Measure	0.52	0.31	327	1.69	0.091	
Language:Heritage:AoA	0.25	0.17	327	1.42	0.157	
Language:Measure:AoA	0.12	0.16	327	0.74	0.460	
Heritage:Measure:AoA	−0.09	0.17	327	−0.53	0.597	
Language:Heritage:Immersion	0.00	0.15	327	0.01	0.994	
Language:Measure:Immersion	−0.14	0.14	327	−0.97	0.333	
Heritage:Measure:Immersion	0.10	0.15	327	0.66	0.511	
Language:Heritage:Family	0.05	0.13	327	0.41	0.680	
Language:Measure:Family	0.23	0.14	327	1.72	0.087	
Heritage:Measure:Family	−0.05	0.13	327	−0.41	0.684	
Language:Heritage:Friends	0.04	0.16	327	0.23	0.815	
Language:Measure:Friends	−0.03	0.15	327	−0.20	0.843	
Heritage:Measure:Friends	0.12	0.16	327	0.76	0.445	
Language:Heritage:Media	−0.25	0.16	327	−1.59	0.112	
Language:Measure:Media	0.04	0.15	327	0.29	0.773	
Heritage:Measure:Media	−0.09	0.16	327	−0.56	0.578	
Language:Heritage:Reading	−0.90	0.16	327	−5.52	<0.001	***
Language:Measure:Reading	−0.38	0.16	327	−2.40	0.017	*
Heritage:Measure:Reading	0.23	0.16	327	1.44	0.150	
Language:Heritage:Individual	0.56	0.18	327	3.15	0.002	**
Language:Measure:Individual	0.10	0.17	327	0.59	0.554	
Heritage:Measure:Individual	−0.04	0.18	327	−0.23	0.817	
Language:Heritage:Measure:AoA	0.54	0.35	327	1.55	0.123	
Language:Heritage:Measure:Immersion	−0.09	0.30	327	−0.31	0.755	
Language:Heritage:Measure:Family	−0.05	0.27	327	−0.20	0.844	
Language:Heritage:Measure:Friends	−0.08	0.31	327	−0.24	0.807	
Language:Heritage:Measure:Media	0.23	0.32	327	0.74	0.462	
Language:Heritage:Measure:Reading	−0.47	0.33	327	−1.45	0.147	
Language:Heritage:Measure:Individual	−0.36	0.36	327	−1.02	0.310	

* p < 0.05

** p < 0.01

*** p < 0.001.

**TABLE 3:** Effects of English experience on self-reported heritage language and English proficiency.

	*Estimate*	*SE*	*df*	*t*	*p*	

(Intercept)	8.70	0.09	110	99.15	<0.001	***
Language	1.71	0.08	330	20.53	<0.001	***
Heritage group	−0.42	0.18	110	−2.38	0.019	*
Measure	−0.10	0.08	330	−1.18	0.237	
AoA	0.14	0.09	110	1.53	0.129	
Immersion	0.06	0.11	110	0.51	0.612	
Family	−0.19	0.10	110	−2.01	0.047	*
Friends	0.01	0.10	110	0.10	0.917	
Media	0.03	0.11	110	0.24	0.807	
Reading	0.06	0.12	110	0.49	0.624	
Individual	−0.12	0.09	110	−1.32	0.191	
Language:Heritage	0.87	0.17	330	5.16	<0.001	***
Language:Measure	0.34	0.17	330	2.05	0.041	*
Heritage:Measure	−0.40	0.17	330	−2.35	0.019	*
Language:AoA	−0.35	0.09	330	−3.99	<0.001	***
Heritage:AoA	0.40	0.18	110	2.17	0.032	*
Measure:AoA	0.05	0.09	330	0.61	0.541	
Language:Immersion	0.14	0.11	330	1.30	0.193	
Heritage:Immersion	0.59	0.24	110	2.47	0.015	*
Measure:Immersion	−0.06	0.11	330	−0.57	0.570	
Language:Family	0.23	0.09	330	2.53	0.012	*
Heritage:Family	−0.22	0.20	110	−1.08	0.283	
Measure:Family	0.11	0.09	330	1.22	0.225	
Language:Friends	−0.17	0.09	330	−1.82	0.069	~
Heritage:Friends	0.21	0.20	110	1.09	0.277	
Measure:Friends	0.00	0.09	330	0.03	0.979	
Language:Media	0.13	0.11	330	1.19	0.235	
Heritage:Media	−0.24	0.22	110	−1.11	0.271	
Measure:Media	−0.07	0.11	330	−0.68	0.499	
Language:Reading	0.27	0.11	330	2.41	0.016	*
Heritage:Reading	−0.03	0.22	110	−0.16	0.874	
Measure:Reading	0.10	0.11	330	0.89	0.373	
Language:Individual	0.08	0.09	330	0.93	0.353	
Heritage:Individual	0.13	0.19	110	0.67	0.507	
Measure:Individual	−0.06	0.09	330	−0.76	0.449	
Language:Heritage:Measure	0.65	0.34	330	1.93	0.054	~
Language:Heritage:AoA	−0.35	0.18	330	−2.00	0.046	*
Language:Measure:AoA	−0.02	0.18	330	−0.12	0.906	
Heritage:Measure:AoA	0.02	0.18	330	0.12	0.907	
Language:Heritage:Immersion	−0.36	0.23	330	−1.57	0.118	
Language:Measure:Immersion	0.14	0.22	330	0.65	0.515	
Heritage:Measure:Immersion	−0.23	0.23	330	−1.01	0.313	
Language:Heritage:Family	0.42	0.19	330	2.19	0.029	*
Language:Measure:Family	−0.07	0.18	330	−0.39	0.697	
Heritage:Measure:Family	0.00	0.19	330	0.00	0.996	
Language:Heritage:Friends	−0.19	0.19	330	−0.99	0.323	
Language:Measure:Friends	−0.07	0.18	330	−0.36	0.721	
Heritage:Measure:Friends	0.25	0.19	330	1.36	0.173	
Language:Heritage:Media	0.20	0.21	330	0.94	0.350	
Language:Measure:Media	0.11	0.21	330	0.50	0.619	
Heritage:Measure:Media	−0.08	0.21	330	−0.40	0.692	
Language:Heritage:Reading	−0.10	0.21	330	−0.48	0.632	
Language:Measure:Reading	−0.08	0.22	330	−0.38	0.704	
Heritage:Measure:Reading	−0.16	0.21	330	−0.78	0.434	
Language:Heritage:Individual	−0.11	0.18	330	−0.61	0.539	
Language:Measure:Individual	−0.05	0.17	330	−0.29	0.772	
Heritage:Measure:Individual	0.16	0.18	330	0.89	0.375	
Language:Heritage:Measure:AoA	−0.19	0.35	330	−0.54	0.592	
Language:Heritage:Measure:Immersion	0.09	0.46	330	0.20	0.845	
Language:Heritage:Measure:Family	0.00	0.38	330	−0.01	0.994	
Language:Heritage:Measure:Friends	−0.46	0.37	330	−1.23	0.221	
Language:Heritage:Measure:Media	0.33	0.42	330	0.79	0.430	
Language:Heritage:Measure:Reading	0.10	0.42	330	0.24	0.807	
Language:Heritage:Measure:Individual	−0.50	0.36	330	−1.38	0.169	

~ p < 0.08

* p < 0.05

*** p < 0.001.

**TABLE 4:** Effects of relative language experience (English—HL) on self-reported heritage language and English proficiency.

	*Estimate*	*SE*	*df*	*t*	*p*	

(Intercept)	8.71	0.07	109	121.64	<0.001	***
Language	1.67	0.06	327	27.14	<0.001	***
Heritage	−0.45	0.14	109	−3.12	0.002	**
Measure	−0.05	0.06	327	−0.84	0.400	
AoA	0.13	0.08	109	1.60	0.113	
Immersion	0.16	0.09	109	1.81	0.072	~
Family	−0.16	0.08	109	−2.05	0.043	*
Friends	−0.01	0.09	109	−0.11	0.913	
Media	−0.04	0.08	109	−0.47	0.637	
Reading	−0.05	0.09	109	−0.60	0.547	
Individual	−0.03	0.08	109	−0.36	0.717	
Language:Heritage	0.65	0.12	327	5.22	<0.001	***
Language:Measure	0.29	0.12	327	2.32	0.021	*
Heritage:Measure	−0.35	0.12	327	−2.79	0.006	**
Language:AoA	−0.22	0.07	327	−3.01	0.003	**
Heritage:AoA	0.37	0.17	109	2.21	0.030	*
Measure:AoA	0.06	0.07	327	0.87	0.384	
Language:Immersion	0.12	0.08	327	1.61	0.108	
Heritage:Immersion	0.55	0.19	109	2.96	0.004	**
Measure:Immersion	−0.06	0.08	327	−0.81	0.416	
Language:Family	0.22	0.07	327	3.33	0.001	**
Heritage:Family	−0.23	0.16	109	−1.48	0.141	
Measure:Family	0.11	0.07	327	1.63	0.104	
Language:Friends	−0.03	0.07	327	−0.39	0.693	
Heritage:Friends	0.33	0.18	109	1.88	0.062	~
Measure:Friends	0.04	0.07	327	0.48	0.630	
Language:Media	0.20	0.07	327	2.82	0.005	**
Heritage:Media	−0.47	0.17	109	−2.76	0.007	**
Measure:Media	−0.04	0.07	327	−0.54	0.587	
Language:Reading	0.35	0.07	327	4.82	<0.001	***
Heritage:Reading	−0.44	0.17	109	−2.62	0.010	*
Measure:Reading	−0.04	0.07	327	−0.59	0.553	
Language:Individual	0.17	0.07	327	2.34	0.020	*
Heritage:Individual	0.18	0.18	109	1.00	0.317	
Measure:Individual	−0.10	0.07	327	−1.41	0.159	
Language:Heritage:Measure	0.54	0.25	327	2.16	0.031	*
Language:Heritage:AoA	−0.52	0.14	327	−3.60	<0.001	***
Language:Measure:AoA	0.00	0.14	327	0.03	0.975	
Heritage:Measure:AoA	0.07	0.14	327	0.49	0.621	
Language:Heritage:Immersion	−0.39	0.16	327	−2.41	0.016	*
Language:Measure:Immersion	0.23	0.16	327	1.48	0.140	
Heritage:Measure:Immersion	−0.27	0.16	327	−1.69	0.091	
Language:Heritage:Family	0.37	0.13	327	2.74	0.007	**
Language:Measure:Family	−0.12	0.13	327	−0.89	0.375	
Heritage:Measure:Family	−0.02	0.13	327	−0.15	0.880	
Language:Heritage:Friends	−0.24	0.15	327	−1.61	0.109	
Language:Measure:Friends	−0.11	0.15	327	−0.73	0.465	
Heritage:Measure:Friends	0.06	0.15	327	0.42	0.673	
Language:Heritage:Media	0.39	0.15	327	2.68	0.008	**
Language:Measure:Media	0.03	0.15	327	0.21	0.831	
Heritage:Measure:Media	−0.08	0.15	327	−0.53	0.597	
Language:Heritage:Reading	0.52	0.15	327	3.56	<0.001	***
Language:Measure:Reading	0.09	0.15	327	0.59	0.557	
Heritage:Measure:Reading	−0.09	0.15	327	−0.59	0.555	
Language:Heritage:Individual	0.30	0.16	327	1.93	0.054	~
Language:Measure:Individual	0.11	0.15	327	0.73	0.464	
Heritage:Measure:Individual	−0.09	0.16	327	−0.56	0.579	
Language:Heritage:Measure:AoA	−0.36	0.29	327	−1.26	0.207	
Language:Heritage:Measure:Immersion	0.26	0.32	327	0.82	0.412	
Language:Heritage:Measure:Family	0.02	0.27	327	0.09	0.932	
Language:Heritage:Measure:Friends	−0.20	0.30	327	−0.66	0.512	
Language:Heritage:Measure:Media	0.15	0.29	327	0.53	0.600	
Language:Heritage:Measure:Reading	0.26	0.29	327	0.88	0.378	
Language:Heritage:Measure:Individual	0.10	0.31	327	0.33	0.743	

~ p < 0.08

* p < 0.05

** p < 0.01

*** p < 0.001.

**TABLE 5:** Effects of heritage language experience on heritage language and English cultural identification.

	*Estimate*	*SE*	*df*	*t*	*p*	

(Intercept)	7.20	0.22	58	32.38	<0.001	***
Language	−0.96	0.44	58	−2.16	0.035	*
Heritage group	−0.33	0.46	58	−0.71	0.480	
Expressive proficiency	0.55	0.28	58	1.95	0.056	~
Receptive proficiency	−0.11	0.34	58	−0.33	0.746	
AoA	0.24	0.23	58	1.04	0.303	
Immersion	−0.28	0.21	58	−1.32	0.191	
Family	0.07	0.22	58	0.31	0.758	
Friends	0.34	0.24	58	1.42	0.161	
Media	−0.04	0.23	58	−0.17	0.862	
Reading	−0.25	0.25	58	−1.00	0.321	
Individual	0.28	0.26	58	1.08	0.284	
Language:Heritage	0.27	0.92	58	0.30	0.765	
Language:Expressive	0.47	0.56	58	0.83	0.410	
Heritage:Expressive	−0.17	0.60	58	−0.28	0.783	
Language:Receptive	−1.29	0.68	58	−1.88	0.065	~
Heritage:Receptive	0.76	0.73	58	1.04	0.302	
Language:AoA	0.13	0.47	58	0.27	0.786	
Heritage:AoA	1.27	0.45	58	2.83	0.006	**
Language:Immersion	−0.12	0.42	58	−0.29	0.777	
Heritage:Immersion	−0.36	0.42	58	−0.85	0.400	
Language:Family	1.02	0.44	58	2.34	0.023	*
Heritage:Family	−0.94	0.47	58	−2.01	0.050	~
Language:Friends	−0.09	0.47	58	−0.19	0.854	
Heritage:Friends	0.59	0.50	58	1.19	0.238	
Language:Media	−0.48	0.46	58	−1.04	0.302	
Heritage:Media	−0.65	0.47	58	−1.39	0.169	
Language:Reading	0.47	0.51	58	0.92	0.361	
Heritage:Readinsg	−0.43	0.49	58	−0.87	0.388	
Language:Individual	−0.01	0.52	58	−0.02	0.988	
Heritage:Individual	−0.13	0.51	58	−0.25	0.802	
Language:Heritage:Expressive	−0.43	1.19	58	−0.37	0.716	
Language:Heritage:Receptive	3.46	1.45	58	2.39	0.020	*
Language:Heritage:AoA	1.40	0.89	58	1.56	0.124	
Language:Heritage:Immersion	−1.31	0.84	58	−1.57	0.122	
Language:Heritage:Family	−1.45	0.93	58	−1.57	0.123	
Language:Heritage:Friends	0.71	0.99	58	0.72	0.477	
Language:Heritage:Media	−0.34	0.93	58	−0.37	0.715	
Language:Heritage:Reading	−2.03	0.98	58	−2.08	0.042	*
Language:Heritage:Individual	1.76	1.01	58	1.74	0.086	

~ p < 0.08

* p < 0.05

** p < 0.01

*** p < 0.001.

**TABLE 6:** Effects of relative language experience (English—HL) on heritage language and English cultural identification.

	*Estimate*	*SE*	*df*	*t*	*p*	

(Intercept)	7.31	0.22	116	33.05	<0.001	***
Language	−0.86	0.44	116	−1.95	0.053	~
Heritage	−0.41	0.46	116	−0.90	0.369	
Expressive proficiency	−0.29	0.31	116	−0.94	0.352	
Receptive proficiency	0.07	0.40	116	0.17	0.867	
AoA	0.12	0.24	116	0.51	0.611	
Immersion	0.64	0.27	116	2.39	0.019	*
Family	0.03	0.25	116	0.14	0.888	
Friends	−0.24	0.25	116	−0.93	0.352	
Media	0.06	0.25	116	0.23	0.815	
Reading	0.21	0.24	116	0.89	0.376	
Individual	−0.10	0.23	116	−0.45	0.657	
Language:Heritage	0.24	0.92	116	0.26	0.796	
Language:Expressive	−0.22	0.61	116	−0.36	0.717	
Heritage:Expressive	0.12	0.63	116	0.18	0.856	
Language:Receptive	1.15	0.79	116	1.45	0.151	
Heritage:Receptive	−1.15	0.84	116	−1.37	0.175	
Language:AoA	0.12	0.47	116	0.26	0.794	
Heritage:AoA	0.11	0.48	116	0.24	0.811	
Language:Immersion	0.46	0.53	116	0.86	0.393	
Heritage:Immersion	0.77	0.55	116	1.41	0.163	
Language:Family	−0.69	0.49	116	−1.40	0.165	
Heritage:Family	1.20	0.51	116	2.34	0.021	*
Language:Friends	0.21	0.51	116	0.42	0.678	
Heritage:Friends	−0.39	0.53	116	−0.72	0.470	
Language:Media	0.24	0.50	116	0.49	0.627	
Heritage:Media	0.58	0.50	116	1.15	0.252	
Language:Reading	−0.31	0.47	116	−0.66	0.509	
Heritage:Reading	0.18	0.47	116	0.37	0.709	
Language:Individual	0.32	0.46	116	0.70	0.486	
Heritage:Individual	−0.24	0.46	116	−0.53	0.598	
Language:Heritage:Expressive	0.79	1.27	116	0.62	0.534	
Language:Heritage:Receptive	−3.50	1.69	116	−2.07	0.041	*
Language:Heritage:AoA	−0.65	0.96	116	−0.68	0.499	
Language:Heritage:Immersion	0.87	1.10	116	0.79	0.430	
Language:Heritage:Family	0.80	1.02	116	0.78	0.435	
Language:Heritage:Friends	−1.35	1.07	116	−1.27	0.208	
Language:Heritage:Media	0.39	1.00	116	0.39	0.697	
Language:Heritage:Reading	1.59	0.94	116	1.69	0.093	
Language:Heritage:Individual	−0.33	0.92	116	−0.36	0.720	

~ p < 0.08

* p < 0.05

*** p < 0.001.

## Data Availability

The dataset analyzed for this study is available from the corresponding author upon reasonable request.
