# Supplementary material for: Predictors of language proficiency and cultural identification in heritage bilinguals
Source: Front Commun (Lausanne). Author manuscript; Available in PMC 2023 Feb 10. (PMC9912478; doi:10.3389/fcomm.2022.994709)
Supplement: Supplementary materials [file NIHMS1862695-supplement-Supplementary_materials.docx]

**Supplementary Materials**

**Table S1. Effects of English Language Experience on Cultural Identification with the Heritage Language and English**

|  | *Estimate* | *SE* | *df* | *t* | *p* |  |
| --- | --- | --- | --- | --- | --- | --- |
| (Intercept) | 7.20 | 0.23 | 116 | 31.50 | < .001 | *** |
| Language | -1.14 | 0.46 | 116 | -2.50 | 0.014 | * |
| Heritage Group | -0.24 | 0.47 | 116 | -0.52 | 0.604 |  |
| Expressive Proficiency | 0.03 | 0.50 | 116 | 0.06 | 0.951 |  |
| Receptive Proficiency | 0.31 | 0.56 | 116 | 0.54 | 0.588 |  |
| AoA | 0.21 | 0.25 | 116 | 0.81 | 0.421 |  |
| Immersion | 0.60 | 0.35 | 116 | 1.69 | 0.094 |  |
| Family | -0.16 | 0.29 | 116 | -0.56 | 0.578 |  |
| Friends | 0.39 | 0.28 | 116 | 1.39 | 0.166 |  |
| Media | -0.10 | 0.30 | 116 | -0.32 | 0.750 |  |
| Reading | -0.20 | 0.38 | 116 | -0.52 | 0.605 |  |
| Individual | -0.07 | 0.29 | 116 | -0.22 | 0.823 |  |
| Language:Heritage | 0.87 | 0.94 | 116 | 0.93 | 0.356 |  |
| Language:Expressive | 0.11 | 1.00 | 116 | 0.11 | 0.913 |  |
| Heritage:Expressive | 0.00 | 0.99 | 116 | 0.00 | 0.999 |  |
| Language:Receptive | 0.24 | 1.13 | 116 | 0.21 | 0.832 |  |
| Heritage:Receptive | 0.29 | 1.16 | 116 | 0.25 | 0.800 |  |
| Language:AoA | 0.01 | 0.51 | 116 | 0.02 | 0.980 |  |
| Heritage:AoA | 0.64 | 0.53 | 116 | 1.22 | 0.226 |  |
| Language:Immersion | 0.18 | 0.71 | 116 | 0.26 | 0.798 |  |
| Heritage:Immersion | 0.49 | 0.73 | 116 | 0.67 | 0.505 |  |
| Language:Family | -0.17 | 0.58 | 116 | -0.30 | 0.764 |  |
| Heritage:Family | 0.87 | 0.61 | 116 | 1.43 | 0.156 |  |
| Language:Friends | 0.57 | 0.55 | 116 | 1.03 | 0.304 |  |
| Heritage:Friends | -0.33 | 0.59 | 116 | -0.57 | 0.571 |  |
| Language:Media | -0.06 | 0.60 | 116 | -0.10 | 0.924 |  |
| Heritage:Media | -0.34 | 0.63 | 116 | -0.55 | 0.584 |  |
| Language:Reading | -0.23 | 0.76 | 116 | -0.30 | 0.767 |  |
| Heritage:Reading | 0.10 | 0.84 | 116 | 0.12 | 0.907 |  |
| Language:Individual | 0.13 | 0.59 | 116 | 0.22 | 0.828 |  |
| Heritage:Individual | -0.75 | 0.62 | 116 | -1.22 | 0.227 |  |
| Language:Heritage:Expressive | 1.83 | 1.97 | 116 | 0.93 | 0.356 |  |
| Language:Heritage:Receptive | -1.11 | 2.32 | 116 | -0.48 | 0.634 |  |
| Language:Heritage:AoA | -0.07 | 1.05 | 116 | -0.07 | 0.946 |  |
| Language:Heritage:Immersion | 0.58 | 1.46 | 116 | 0.39 | 0.695 |  |
| Language:Heritage:Family | 0.55 | 1.22 | 116 | 0.46 | 0.649 |  |
| Language:Heritage:Friends | -1.42 | 1.17 | 116 | -1.21 | 0.227 |  |
| Language:Heritage:Media | -0.19 | 1.25 | 116 | -0.15 | 0.880 |  |
| Language:Heritage:Reading | 1.13 | 1.67 | 116 | 0.68 | 0.499 |  |
| Language:Heritage:Individual | -0.31 | 1.23 | 116 | -0.25 | 0.803 |  |

## *Note*. ~*p <* .08, **p <* .05, ***p <* .01, ****p <* .001

**Supplemental Analyses for Effects of English AoA and English Family on Heritage Language Proficiency**

Supplemental analyses examined whether effects of English AoA and English acquisition and exposure through family on HL proficiency were moderated by overall HL experience. A measure of overall HL experience was calculated by aggregating across ratings of HL acquisition and exposure through family, friends, media, reading, and individual instruction. Overall HL experience was significantly higher among Spanish bilinguals (*M* = 3.40) than non-Spanish bilinguals (*M* = 2.81), *t*(113.23) = 2.49, *p* = 0.014. Receptive and expressive proficiency ratings were entered as the outcome variable in a linear mixed effects model with fixed effects of Language (HL vs. English), Heritage Group (Spanish vs. Non-Spanish), Measure (Expressive vs. Receptive), HL Experience, English AoA, English Family, and all interactions with English AoA and English Family.

A significant interaction emerged between Language, HL Experience, and English AoA (*p* = 0.016), as well as between Language, HL Experience, and English Family (*p* = 0.044; see Table S2 for full output). Tukey-adjusted simple effects revealed that later English AoA was associated with significantly higher HL proficiency among bilinguals with relatively less HL experience (-1 SD from mean: *Estimate* = 0.47, *SE* = 0.13, *t*(150) = 3.65, *p* < .001), but not for those with greater HL experience (+1 SD: *Estimate* = 0.07, *SE* = 0.13, *t*(150) = 0.53, *p* = 0.599). Likewise, greater English acquisition and exposure through family was associated with significantly lower HL proficiency among bilinguals with less HL experience (*Estimate* = -0.48, *SE* = 0.12, *t*(150) = -4.13, *p* < .001), but not among those with greater HL experience (*Estimate* = -0.15, *SE* = 0.14, *t*(150) = -1.01, *p* = 0.312). These effects were not moderated by Heritage Group and neither English AoA nor Family significantly impacted English proficiency (*ps* > .05).

**Table S2. Effects of English AoA and Family on Heritage Language Proficiency by Heritage Group and Heritage Language Experience**

|  | *Estimate* | *SE* | *df* | *t* | *p* |  |
| --- | --- | --- | --- | --- | --- | --- |
| (Intercept) | 8.70 | 0.08 | 106 | 105.83 | <.001 | *** |
| Language | 1.65 | 0.07 | 318 | 22.60 | <.001 | *** |
| Heritage Group | -0.31 | 0.16 | 106 | -1.89 | 0.061 | ~ |
| Measure | -0.04 | 0.07 | 318 | -0.49 | 0.623 |  |
| HL Experience | 0.10 | 0.09 | 106 | 1.01 | 0.313 |  |
| AoA | 0.08 | 0.08 | 106 | 1.03 | 0.305 |  |
| Family | -0.17 | 0.09 | 106 | -2.00 | 0.048 | * |
| Language:Heritage | 0.25 | 0.15 | 318 | 1.73 | 0.084 |  |
| Language:Measure | 0.23 | 0.15 | 318 | 1.55 | 0.123 |  |
| Heritage:Measure | -0.25 | 0.15 | 318 | -1.73 | 0.085 |  |
| Language:HL_Exp | -0.44 | 0.08 | 318 | -5.24 | <.001 | *** |
| Heritage:HL_Exp | 0.23 | 0.19 | 106 | 1.23 | 0.220 |  |
| Measure:HL_Exp | 0.06 | 0.08 | 318 | 0.70 | 0.484 |  |
| Language:AoA | -0.34 | 0.07 | 318 | -4.81 | <.001 | *** |
| Language:Family | 0.28 | 0.08 | 318 | 3.64 | <.001 | *** |
| Heritage:AoA | 0.13 | 0.16 | 106 | 0.80 | 0.424 |  |
| Heritage:Family | 0.02 | 0.18 | 106 | 0.12 | 0.901 |  |
| Measure:AoA | 0.06 | 0.07 | 318 | 0.86 | 0.389 |  |
| Measure:Family | 0.00 | 0.08 | 318 | -0.02 | 0.984 |  |
| HL_Exp:AoA | -0.09 | 0.09 | 106 | -1.07 | 0.287 |  |
| HL_Exp:Family | 0.10 | 0.08 | 106 | 1.30 | 0.196 |  |
| Language:Heritage:Measure | 0.39 | 0.29 | 318 | 1.33 | 0.183 |  |
| Language:Heritage:HL_Exp | -0.43 | 0.17 | 318 | -2.57 | 0.011 | * |
| Language:Measure:HL_Exp | -0.16 | 0.17 | 318 | -0.92 | 0.357 |  |
| Heritage:Measure:HL_Exp | 0.19 | 0.17 | 318 | 1.12 | 0.263 |  |
| Language:Heritage:AoA | -0.14 | 0.14 | 318 | -0.98 | 0.328 |  |
| Language:Heritage:Family | 0.03 | 0.16 | 318 | 0.21 | 0.835 |  |
| Language:Measure:AoA | -0.06 | 0.14 | 318 | -0.44 | 0.663 |  |
| Language:Measure:Family | 0.02 | 0.15 | 318 | 0.16 | 0.873 |  |
| Heritage:Measure:AoA | 0.18 | 0.14 | 318 | 1.24 | 0.215 |  |
| Heritage:Measure:Family | -0.13 | 0.16 | 318 | -0.82 | 0.415 |  |
| Language:HL_Exp:AoA | 0.19 | 0.08 | 318 | 2.41 | 0.016 | * |
| Language:HL_Exp:Family | -0.14 | 0.07 | 318 | -2.02 | 0.044 | * |
| Heritage:HL_Exp:AoA | -0.13 | 0.17 | 106 | -0.78 | 0.434 |  |
| Heritage:HL_Exp:Family | -0.08 | 0.16 | 106 | -0.53 | 0.594 |  |
| Measure:HL_Exp:AoA | -0.05 | 0.08 | 318 | -0.62 | 0.536 |  |
| Measure:HL_Exp:Family | 0.03 | 0.07 | 318 | 0.37 | 0.708 |  |
| Language:Heritage:Measure:HL_Exp | -0.15 | 0.33 | 318 | -0.46 | 0.646 |  |
| Language:Heritage:Measure:AoA | -0.28 | 0.28 | 318 | -0.98 | 0.328 |  |
| Language:Heritage:Measure:Family | -0.06 | 0.32 | 318 | -0.19 | 0.846 |  |
| Language:Heritage:HL_Exp:AoA | 0.16 | 0.15 | 318 | 1.08 | 0.283 |  |
| Language:Heritage:HL_Exp:Family | -0.01 | 0.14 | 318 | -0.05 | 0.958 |  |
| Language:Measure:HL_Exp:AoA | 0.08 | 0.15 | 318 | 0.53 | 0.593 |  |
| Language:Measure:HL_Exp:Family | -0.03 | 0.14 | 318 | -0.22 | 0.822 |  |
| Heritage:Measure:HL_Exp:AoA | 0.05 | 0.15 | 318 | 0.30 | 0.762 |  |
| Heritage:Measure:HL_Exp:Family | -0.13 | 0.14 | 318 | -0.94 | 0.348 |  |
| Language:Heritage:Measure:HL_Exp:AoA | 0.18 | 0.31 | 318 | 0.57 | 0.566 |  |
| Language:Heritage:Measure:HL_Exp:Family | -0.15 | 0.28 | 318 | -0.54 | 0.591 |  |

## *Note*. ~*p <* .08, **p <* .05, ***p <* .01, ****p <* .001
